# Functional analysis of a panel of molecular markers for diagnosis of systemic lupus erythematosus in rats

**DOI:** 10.1042/BSR20240318

**Published:** 2024-07-19

**Authors:** May A. Azzam, Sally A. Fahim, Asmaa A. ElMonier, Nadine W. Maurice

**Affiliations:** 1 Department of Biochemistry, Faculty of Pharmacy, Cairo University, 11562, Cairo, Egypt; 2 Department of Biochemistry, School of Pharmacy, Newgiza University (NGU), Newgiza, Km 22 Cairo-Alexandria Desert Road, 12577, Giza, Egypt

**Keywords:** circ-CDC27, Circ-Med14, circ-TubD1, rno-miR-146a-5p, Systemic lupus erythematosus, TRAF6

## Abstract

Introduction: Systemic lupus erythematosus (SLE) is a diverse autoimmune disease that arises from a combination of complex genetic factors and environmental influences. While circRNAs and miRNAs have recently been identified as promising biomarkers for disease diagnosis, their specific expression patterns, and clinical implications in SLE are not yet fully understood.

Aim of the work: The aim of the present study was to determine the role of a panel of noncoding-RNAs specifically circRNAs (circ-TubD1, circ-CDC27, and circ-Med14), along with miRNA (rno-miR-146a-5p) and mRNA (TRAF6), as novel minimally invasive diagnostic biomarkers for experimentally induced SLE. Additionally, the study involved an insilico bioinformatics analysis to explore potential pathways involved in the pathogenesis of SLE, aiming to enhance our understanding of the disease, enable early diagnosis, and facilitate improved treatment strategies.

Materials and methods: SLE was induced in rats using single IP injection of incomplete Freund’s adjuvant (IFA). The Induction was confirmed by assessing the ANA and anti-ds DNA levels using ELSA technique. qPCR analysis was conducted to assess the expression of selected RNAs in sera collected from a group of 10 rats with induced SLE and a control group of 10 rats. In addition, bioinformatics and functional analysis were used to construct a circRNA–miRNA–mRNA network and to determine the potential function of these differentially expressed circRNAs.

Results: SLE rats demonstrated significantly higher expression levels of circ-CDC27, circ-Med14, and rno-miR-146a-5p as well as TRAF6, with lower expression level of circ-TubD1 in sera of SLE rats relative to controls. ROC curve analysis indicated that all the selected non-coding RNAs could serve as potential early diagnostic markers for SLE. In addition, the expression level of circ-TubD1 was negatively correlated with rno-miR-146a-5p, however, rno-miR-146a-5p was positively correlated with TRAF6. Bioinformatic analysis revealed the incorporation of the circRNAs targeted genes in various immune system and neurodegeneration pathways.

Conclusions: Therefore, circRNAs; circ-TubD1, circ-CDC27, and circ-Med14, in addition to the miRNA (rno-miR-146a-5p) and mRNA (TRAF6) may be involved in the development of SLE and may have promising roles for future diagnosis and targeted therapy.

## Introduction

Systemic lupus erythematosus (SLE) is a chronic autoimmune condition that impacts multiple organs in the body, producing numerous autoantibodies, immune complex deposits, and damaging various body systems. This damage can range from skin lesions, hematological abnormalities, and kidney damage to nervous system involvement and musculoskeletal manifestations [1]. In Egypt, the estimated occurrence of SLE among adult patients was calculated to be 6.1 per 100,000 individuals, whereas the global SLE prevalence was estimated to be 43.7 per 100000 persons [2,3].

Early management and appropriate treatment are crucial in preventing severe clinical manifestations in SLE patients. Previous research has established that SLE arises from the intricate interplay of genetic, epigenetic, and environmental factors [4]. Numerous genome-wide association studies have confirmed known associations and identified new susceptibility genes associated with SLE [5–7]. Therefore, investigating the genetic and molecular abnormalities of SLE is required and necessary for identifying new diagnostic biomarkers.

Tumor necrosis factor receptor (TNFR)-associated factor 6 (TRAF6), a potential gene associated with susceptibility to SLE, functions as a signaling molecule in the TNFR superfamily and the interleukin-1 receptor/Toll-like receptor superfamily. Within the immune system, TRAF6 plays a central role in activating the nuclear factor-κB (NF-κB) pathway. The signaling mediated by TRAF6 has demonstrated its critical importance in the development, maintenance, and activation of various immune cell types, including B cells, T cells, dendritic cells, and macrophages [8,9].

Non-coding RNAs, a group of RNA transcripts that do not encode proteins, have emerged as crucial regulatory molecules in diverse biological processes. Among them, microRNAs (miRNAs), approximately 22–25 nucleotides in length, are a type of non-coding, single-stranded RNA molecules encoded by endogenous genes. They play a role in regulating gene expression at the transcriptional level. The regulatory functions of miRNAs are involved in numerous biological processes, such as individual growth and development, cell proliferation and apoptosis, cell metabolism, cell differentiation, and immune-inflammatory responses [10]. MiR-146a, situated on the long arm of chromosome 5, belongs to the miR-146 family. MiR-146a is classified as an immune- and hematopoiesis-related miRNA, actively involved in processes such as hematopoietic cell proliferation, differentiation, immune cell responses, and the release of inflammatory mediators. Notably, one of its crucial roles is the regulation of the NF-κB signaling pathway activation and inflammatory cytokines by targeting TRAF6. Consequently, dysregulation of miR-146a has been observed in various chronic inflammatory conditions, including SLE, rheumatoid arthritis, osteoarthritis, and psoriasis [10,11].

In contrast, circular RNAs (circRNAs) represent a distinct type of non-coding RNA characterized by their circular structure, primarily derived from exon transcripts that form closed RNA circles. Unlike linear RNAs, circRNAs exhibit greater stability and resistance to being degraded by exonucleases due to the absence of 5′ or 3′ ends. Furthermore, circRNAs often display tissue- or developmental stage-specific expression patterns, making them potential biomarkers compared with linear RNAs. Studies have revealed that circRNAs can act as miRNA sponges or competing endogenous RNAs (ceRNAs), sequestering target miRNAs and modulating the activity of RNA-binding proteins to regulate gene transcription. Extensive evidence suggests that circRNAs may play significant roles in neurological disorders, atherosclerotic vascular disease, cancer, and autoimmune diseases. This supports the notion that circRNAs have the potential to serve as novel diagnostic and prognostic biomarkers and represent promising therapeutic targets for various diseases [12,13]. Interestingly, circ-tubulin delta 1 (circ-TubD1) has been demonstrated to act as miR-146a-5p sponge, affecting pro-inflammatory cytokine production through regulating the expression of TRAF6 in hepatic cell line [7]. Previously, it has been reported that circ-0044235, spliced from cell division cycle 27 (CDC27) gene [12–14], was differentially expressed in patients of SLE when compared with healthy controls. These studies supported the potential use of circ-0044235 as a diagnostic biomarker for SLE, but the results were inconsistent in these studies, where a previous study of Luo et al. [12] demonstrated a decrease in the level of circ-0044235 in peripheral blood leukocytes of SLE patients; however, a significant increase in its level was detected in new-onset SLE. On the other hand, Luo et al. [13] reported a decrease in the level of circ-0044235 in new-onset SLE. In addition, circ-0140271, a circRNA generated from the mediator complex subunit 14 (MED14) gene located on the X-chromosome, was suggested as a potential diagnostic biomarker for the chronic autoimmune disease; rheumatoid arthritis (RA), as it may act as miRNA sponge to regulate fatty acid metabolism pathways in RA [15]. However, to date, the expression of this novel circRNA has not been demonstrated in SLE. Accordingly, we are interested in elucidating the regulatory role of these circRNAs in the development of SLE.

Therefore, the aim of the present study was to determine the role of novel non-invasive diagnostic RNA biomarkers as circRNAs; circ-TubD1 (circ-0044897), circ-CDC27 (circ-0044235), and circ-Med14 (circ-0140271), in addition to the miRNA (rno-miR-146a-5p) and mRNA (TRAF6) in SLE-induced rats. Moreover, bioinformatics and functional analysis were used to construct a network involving circRNA–miRNA–mRNA interaction. This network aimed to shed light on the potential roles and implications of these circRNAs in SLE.

## Materials and methods

### Experimental animals

This study included 20 adult Wistar albino male rats weighing 200 ± 20 g obtained from Theodor Bilharz Research Institute (Giza, Egypt) and the experimental work was performed in School of Pharmacy, Newgiza University. The rats were acclimatized to the experimental conditions for a period of 7 days before initiating the study. During this time, they were provided with ad libitum access to water and standard pellet food. The rats were housed in a well-ventilated animal facility throughout the duration of the study. Animals were divided into two groups; 10 randomly assigned rats received a single 0.5 ml intraperitoneal injection of incomplete Freund’s adjuvant (IFA) for induction of SLE [16]. On the other side, the control group consisted of 10 rats, which received 0.5 ml intraperitoneal injection of saline. After a period of 6 weeks, the animals were hypnotized using thiopental sodium (50 mg/kg, I.P.) [17], and then euthanized by cervical dislocation. Blood samples were collected, and the serum was separated by centrifugation at 13,000 RPM for 10 minutes at room temperature. The collected serum was then stored at −80°C for further biochemical and molecular investigations.

This study was approved by the research committee of School of Pharmacy, Newgiza University (BC-0052). The study methodology adhered to the guidelines set forth by the US National Institute of Health’s Guide for the Care and Use of Laboratory Animals (NIH Publication No 85-23, revised 2011). Every effort was made to minimize animal distress and the number of animals utilized in the study.

### Biochemical analysis

Serum concentrations of antinuclear antibodies (ANA) and anti-double-stranded DNA (anti-dsDNA) antibody were determined using ELISA kits (MyBioSource, U.S.A., Cat. No.: MBS269217 and Cat. No.: MBS727350, respectively). The kits utilize the Double Antigen Sandwich ELISA technique. Samples were added into ELISA plate wells and washed out with PBS after their respective additions to the wells. Then, Avidin-peroxidase conjugates were added to the wells in after. Tetra methyl benzidine (TMB) substrate was used for coloration after the enzyme conjugate has already been thoroughly washed out of the wells by PBS. TMB reacted to form a blue product from the peroxidase activity, and finally turned to yellow after addition of the stop solution. The color intensity and quantity of target analyte in the sample were analyzed using Tecan, A-5082 ELISA reader at wavelength of 450 nm for both markers.

### Estimation of the relative expression levels of the studied circRNAs and TRAF6 mRNA

Circ-CDC27 (circ-0044235), circ-Med14 (circ-0140271), circ-TubD1 (circ-0044897) and TRAF6 were extracted from rats’ sera using RNeasy mini extraction kit (Qiagen, Germany, Cat. No.: 74104) using the manual’s instructions. The RNA’s concentration and purity were then determined at 260/280 nm using Nanodrop® spectrophotometer, U.S.A., with acceptable ratio of > 1.8. Next, these RNAs were reverse transcribed using cDNA reverse transcription kit (Thermo Fisher Scientific, U.S.A., Cat. No.: 4368814) using the thermal cycler, which was programmed as follows: 37°C for 1 h followed by 10 min at 95°C, and finally cooled at 4°C. The resultant cDNA was then stored at −20°C till further analysis. Finally, quantitative real-time polymerase chain reaction (qPCR) was conducted utilizing Step One Real-time PCR system, U.S.A., adjusting it at 50°C for 2 min followed by 40 cycles of 15 s at 95°C, and finally 1 min at 60°C.

### Estimation of the relative expression level of rno-miR-146a-5p

Rno-miR-146a-5p was extracted from rats’ sera using mirvana kit (Thermo Fisher Scientific, U.S.A., Cat. No.: AM1560). The concentration and purity of the resultant RNA was measured as previously mentioned. Next, rno-miR-146a-5p was reverse-transcribed using TaqMan® MicroRNA assay kit (Thermo Fisher Scientific, U.S.A., Cat. No.: 4427975) using the thermal cycler, which was programmed as follows: lead was heated at 112°C to prevent sample loss, then 16°C and 42°C for 30 min each, and finally for 5 min at 85°C. The resultant cDNA was then stored at −20°C till further analysis. Additionally, qPCR amplification was performed using TaqMan® MicroRNA assay kit with the following cycling conditions: 2 min at 50°C for PCR initial heat activation step (one cycle), followed by 40 cycles of 15 s at 95°C, 2 min at 60°C.

### Data analysis

Relative expression levels were determined by the 2^−ΔΔCT^ method using β-actin as a house-keeping reference gene for circ-CDC27 (circ-0044235), circ-Med14 (circ-0140271), circ-TubD1 (circ-0044897), and TRAF6, whereas U6 was used as a reference gene for rno-miR-146a-5p.

The primer sequences employed for PCR amplification of the targeted RNAs are provided in Table 1. All primers we used in the present study have 100% efficiency by using the equation: *E* = -1+10 (-1/slope).

**Table 1:** Primers’ sequences used in qPCR reactions

Gene ID	Primer sequence	Accession number
circ-CDC27 (circ-0044235)	F: 5′-CACCTACCTTCTCACCACTACA-3′	NM_001415702.1
	R: 5′-GGTCGCCAGTAGAAACAAGG-3′	
circ-Med14 (circ-0140271)	F: 5′-ATCCTCCTTTGCCAGCTTCT-3′	NM_001191727.2
	R: 5′-TCCGGCTAGCAAACTGTACA-3′	
circ-TubD1 (circ_0044897)	F: 5′-GTAAGACCCCAGCCTCTGTC-3′	NM_001105826.2
	R:5′-GGGTCTCAGAATTCCAGGTCT-3′	
TRAF6	F: 5′-CAGTCCCCTGCACATT-3′	NM_001107754.2
	R: 5′-GAGGAGGCATCGCAT-3′	
Rno-miR-146a-5p	F: 5′-CGCTACTCGTACCGTGAGTAA-3′	MIMAT0000852
	R: 5′-GTGCAGGGTCCGAGGT-3′	
β-Actin	F: 5′-CAGGGTGTGATGGTGGGTATGG-3′	NM_031144.3
	R: 5′-AGTTGGTGACAATGCCGTGTTC-3′	
U6	F: 5′-CTCGCTTCGGCAGCACATA-3′	XR_005488834.1
	R: 5′-CGCTTCACGAATTTGCGTG-3′	

Abbreviations: CDC27, cell division cycle 27; Med14, mediator complex subunit 14; Traf6, tumor necrosis factor receptor-associated factor 6; TubD1, tubulin delta 1.

### Integrated target prediction of circRNAs’ target genes using bioinformatics analysis

Firstly, the circRNA sequence, accession number and location were retrieved from Circbase database [18] and CircAtlas [19], NCBI blast was used to assure the conservation of the circRNAs between species. Then, the target miRNAs were obtained from Targetscan 8.0 database [20], miRanda V3.3a software, and CircAtlas [19]. The common miRNAs were chosen for further analysis.

The target genes of the chosen miRNAs were predicted using Targetscan V8.0 [20], miRDB [21], and pictar [22]. The intersection between these targets’ genes was identified by a Venn diagram. The interaction network was represented using Cytoscape V3.10.

### The functions of circRNAs’ target genes using bioinformatics

The 515, 267, 210 overlapping genes of Circ-CDC27, Circ-Med14, and Circ-TubD1 (circ-0044897) were then submitted to David [23] and ShinyGO V0.80 [24] for gene ontology (GO) annotation and functional enrichment analyses including KEGG, Wikipathways, Reactome, BioCarta, and Canonical pathways enrichment analysis were also performed. *P*<0.05 was considered significant.

### Statistical analysis

Normal distribution of the data was assessed using the Kolmogorov–Smirnov test. Data werexpressed as mean ± S.D and Student’s *t*-test was used for two-group comparisons. The diagnostic accuracy of RNA levels in the sera of SLE-induced rats was assessed using ROC curve. Pearson correlation test was employed to investigate potential linear associations between the expression levels of the analyzed RNAs. A *P*-value of less than 0.05 was considered statistically significant. All statistical analyses were conducted using GraphPad Prism 5.0 software.

## Results

### Biochemical assessment

**Figure 1:**
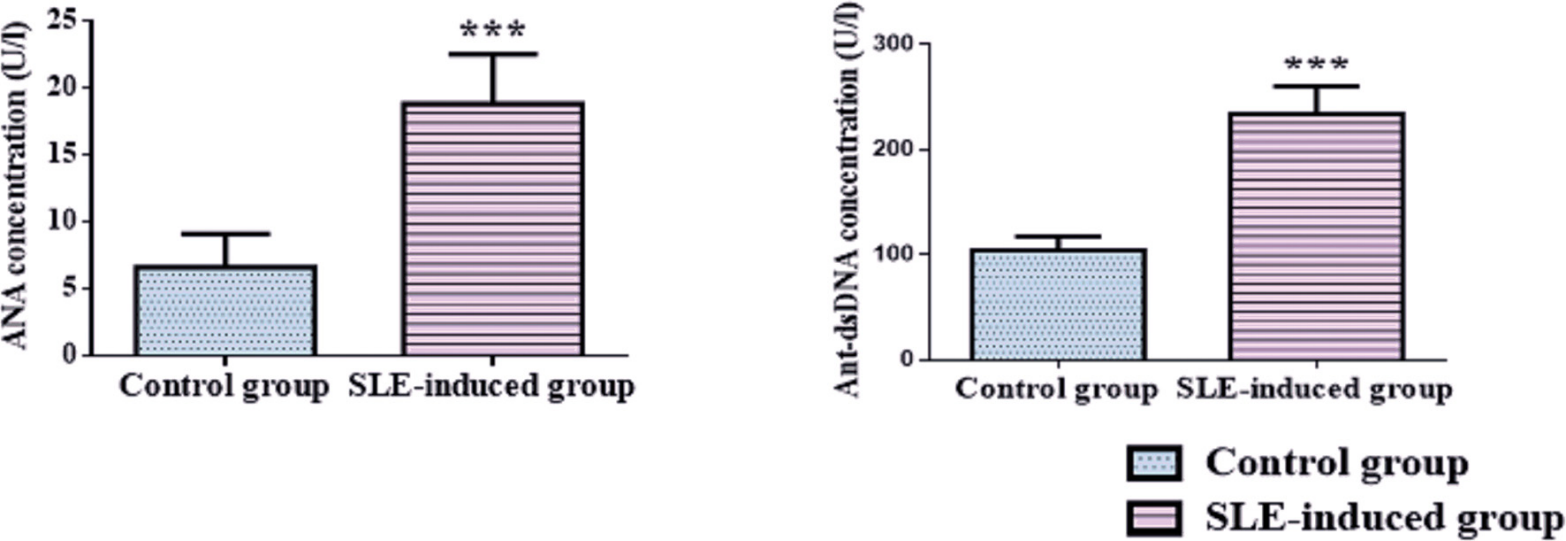
Concentration of ANA and anti-dsDNA measured in sera of rats following induction of SLE Data are represented as mean ± S.D by bar chart. ANA, anti-nuclear antibody; Anti-dsDNA, anti-double stranded DNA; SLE, systemic lupus erythematosus. Significantly different at ***P<0.001. Data were analyzed by Student’s *t*-test.

There was a strong elevation in ANA and anti-dsDNA levels confirming induction of SLE when measured in rats’ sera at *P*<0.001 compared with the control group as demonstrated in Figure 1 and Supplementary Table S1.

### Relative expression levels of the studied RNAs in sera of SLE-induced rats

**Figure 2:**
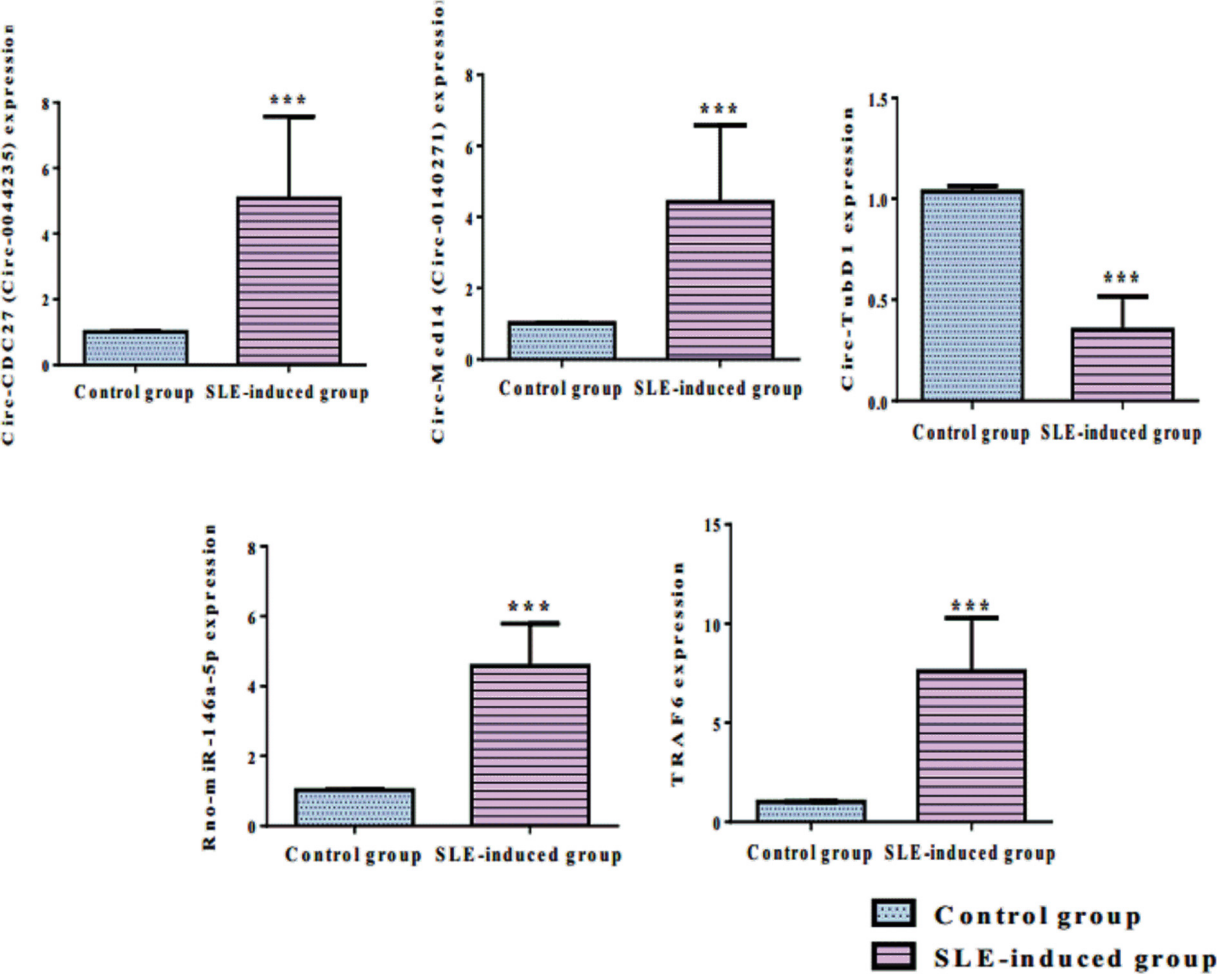
Expression levels of the studied RNAs following induction of SLE in sera of rats Data are represented as mean ± S.D. by bar chart. CDC27, cell division cycle 27; Med14, mediator complex subunit 14; SLE, systemic lupus erythematosus; TubD1, tubulin delta 1. Significantly different at ***P<0.001. Data were analyzed by Student’s *t*-test.

In comparison with controls, SLE rats demonstrated significantly higher expression levels of circ-CDC27 (circ-0044235), circ-Med14 (circ-0140271), and rno-miR-146a-5p as well as the mRNA of TRAF6, with lower expression level of circ-TubD1 (circ-0044897) reaching about 5.08-, 4.42-, 4.58-, 7.6-, and 0.35-fold of control values at *P*<0.001 as depicted in Figure 2 and Supplementary Table S2.

### Diagnostic performance of the studied circRNAs and rno-miR-146a-5p

**Figure 3:**
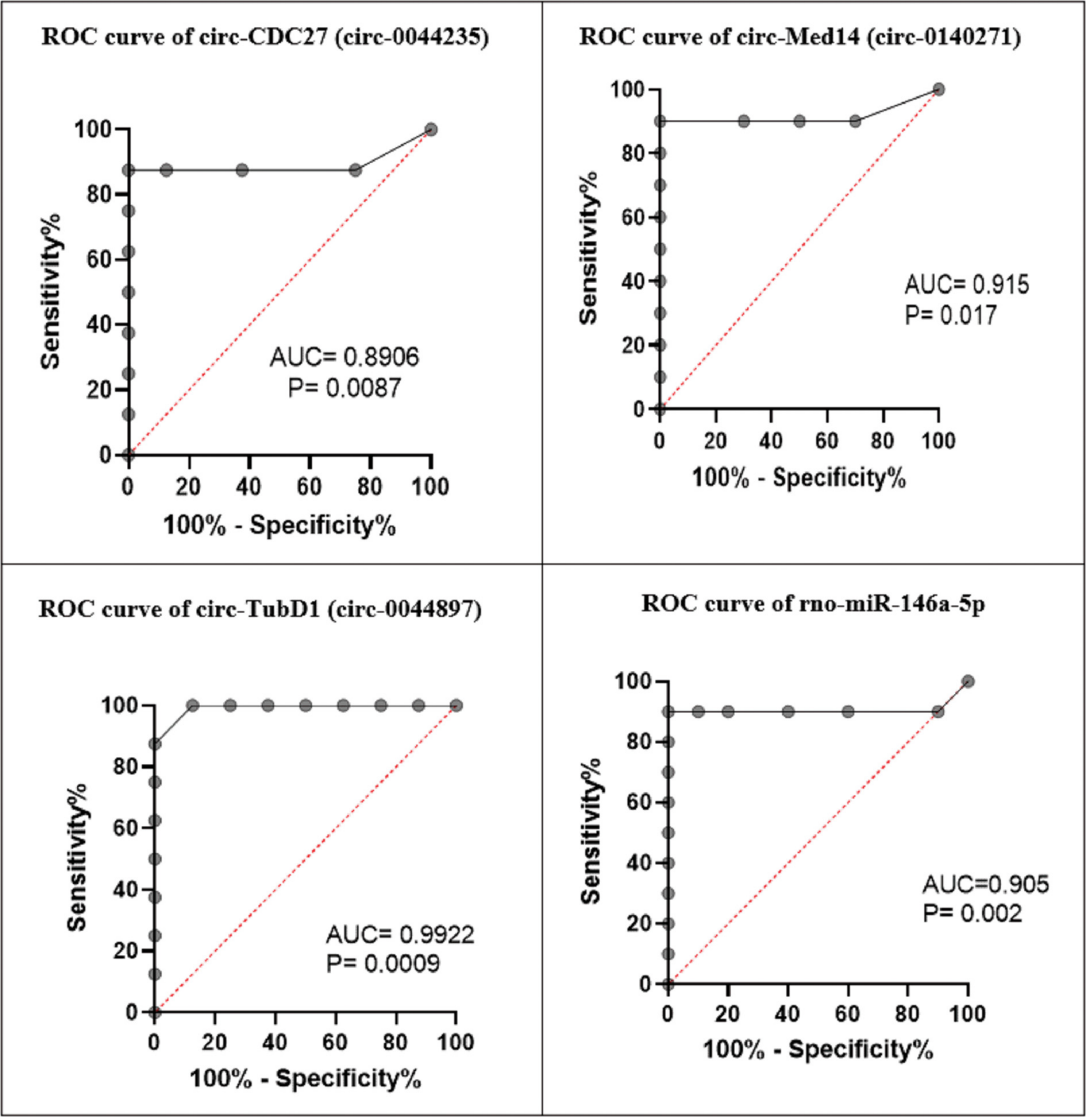
Receiver operating characteristic curve analysis displaying the diagnostic power of studied non-coding RNAs measured in sera of SLE-induced rats AUC, area under the curve; CDC27, cell division cycle 27; Med14, mediator complex subunit 14; TubD1, tubulin delta 1. Significant at *P*-values < 0.05.

To evaluate the diagnostic potential and discriminatory accuracy of previously mentioned markers, ROC curve analysis was performed. The analysis revealed that the circ-RNAs: circ-CDC27 (circ-00442315), circ-Med14 (circ-0140271), circ-TubD1 (circ-0044897), and rno-miR-146a-5p had the potential to discriminate rats with SLE from control group with AUC of 0.9922 (*P*=0.0009), 0.8906 (*P*=0.008), 0.915 (*P*=0.017), and 0.905 (*P*=0.002), respectively, as shown in Figure 3.

### Correlation

**Figure 4:**
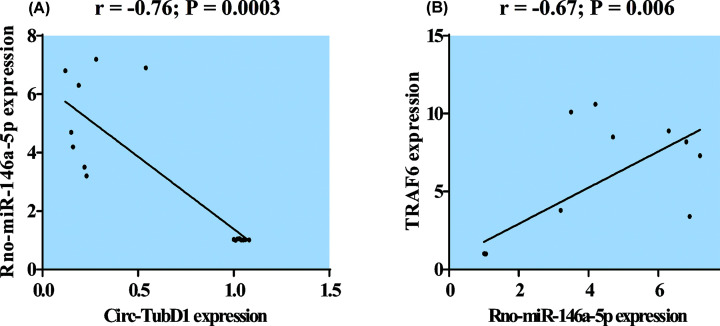
Pearson correlation analysis of circ-TubD1 and rno-miR-146a-5p (A), rno-miR-146a-5p and TRAF6 (B) *r*, correlation coefficient; TRAF6, tumor necrosis factor receptor-associated factor 6; TubD1, tubulin delta 1. Significant at *P*-values < 0.05.

The relationship between the relative expression levels of cir-TubD1, rno-miR-146a-5p, and TRAF6 were explored and showed that the expression level of circ-TubD1 (circ-0044897) was negatively correlated with rno-miR-146a-5p (*r* = −0.76; *P*=0.0003), as shown in Figure 4A. However, rno-miR-146a-5p was positively correlated with TRAF6 (*r* = 0.67; *P*=0.006), as shown in Figure 4B.

Correlating ANA and anti-dsDNA with the biomarkers under investigation showed that the levels of circ-Med14 and rno-miR-146a-5p were strongly positively correlated with both ANA and anti-dsDNA levels, respectively (circ-Med14 vs ANA: *r* = 0.97, *P*=0.0001; rno-miR-146a-5p vs anti-dsDNA: *r* = 0.89, *P*=0.0009) as shown in Supplementary Figure S1.

### Integrated targets prediction of circ-CDC27, circ-Med14, and CircTubD1

**Figure 5:**
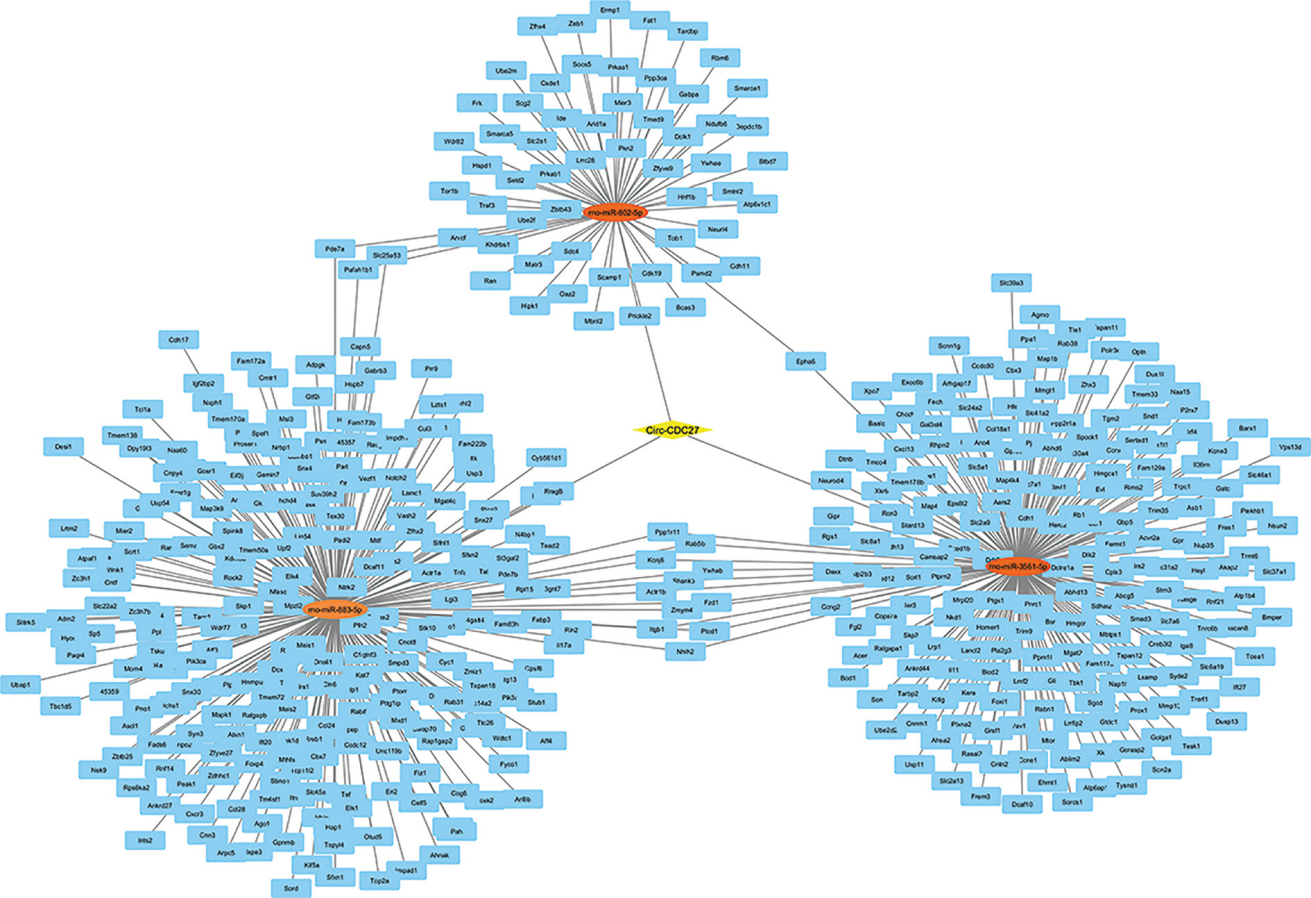
The circRNA–miRNA–mRNA interaction network constructed with cytoscape The network consists of Circ-CDC27 (yellow node), miRNAs (red), and their target genes (blue). The target miRNAs were obtained from the intersection between Targetscan, miRanda, and CircAtlas databases. The 515 target genes of the chosen miRNAs were predicted using Targetscan, miRDB, and pictar databases. CDC27, cell division cycle 27.

**Figure 6:**
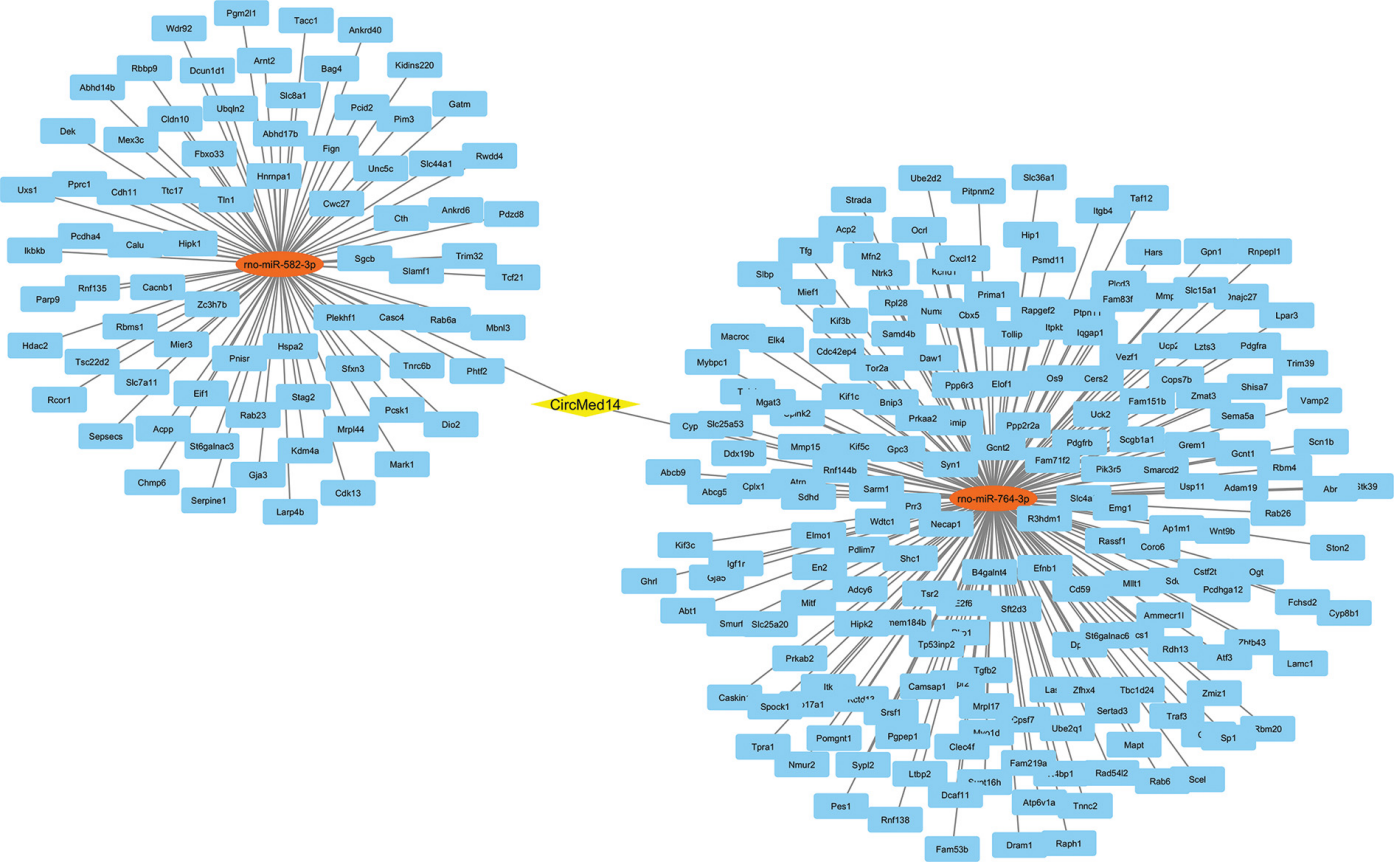
The circRNA–miRNA–mRNA interaction network constructed with cytoscape The network consists of Circ-Med14 (yellow node), miRNAs (red), and their target genes (blue). The target miRNAs were obtained from the intersection between Targetscan, miRanda, and CircAtlas databases. The 267 target genes of the chosen miRNAs were predicted using Targetscan, miRDB, and pictar databases. Med14, mediator complex subunit 14.

**Figure 7:**
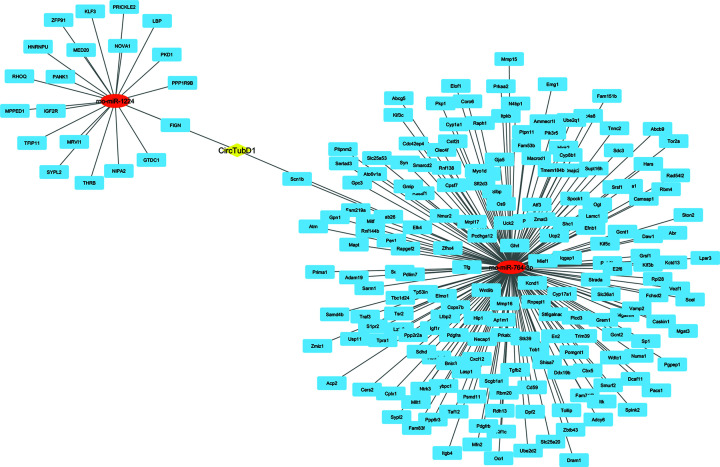
The circRNA-miRNA-mRNA interaction network constructed with cytoscape. The network consists of Circ-TubD1 (yellow node), miRNAs (red), and their target genes (blue) The target miRNAs were obtained from the intersection between Targetscan, miRanda, and CircAtlas databases. The 210 target genes of the chosen miRNAs were predicted using Targetscan, miRDB, and pictar databases. TubD1, tubulin delta 1.

CircRNAs can modulate gene expression by impacting various processes such as transcription, mRNA degradation, and translation. They achieve this by acting as molecular sponges for RNA-binding proteins and miRNAs [25]. MiRNAs interact with the 3′ untranslated region (3′UTR) of target genes, resulting in the suppression of their translation process [26]. Using Targetscan, miRanda, and CircAtlas and the venn diagram to determine the intersection of the adsorbable miRNAs, Circ-CDC27 was predicted to sponge rno-miR-883-5p, rno-miR-802-5p, and rno-miR-3561-5p (Figure 5), while CircMed-14 adsorbed rno-miR-582-3p and rno-miR-764-3p (Figure 6), and Circ-TubD1 (circ-0044897) was correlated to rno-miR-1224 and rno-miR-764-3p (Figure 7).

To understand the core function of miRNAs, its target genes were identified. A total of 515, 267, 210 overlapping genes of the sponged miRNAs of Circ-CDC27, Circ-Med14, and Circ-TubD1 (circ-0044897), respectively, were predicted with high potential using three additional miRNA-target prediction tools.

### Functional enrichment analysis and GO annotation of the predicted target genes

**Figure 8:**
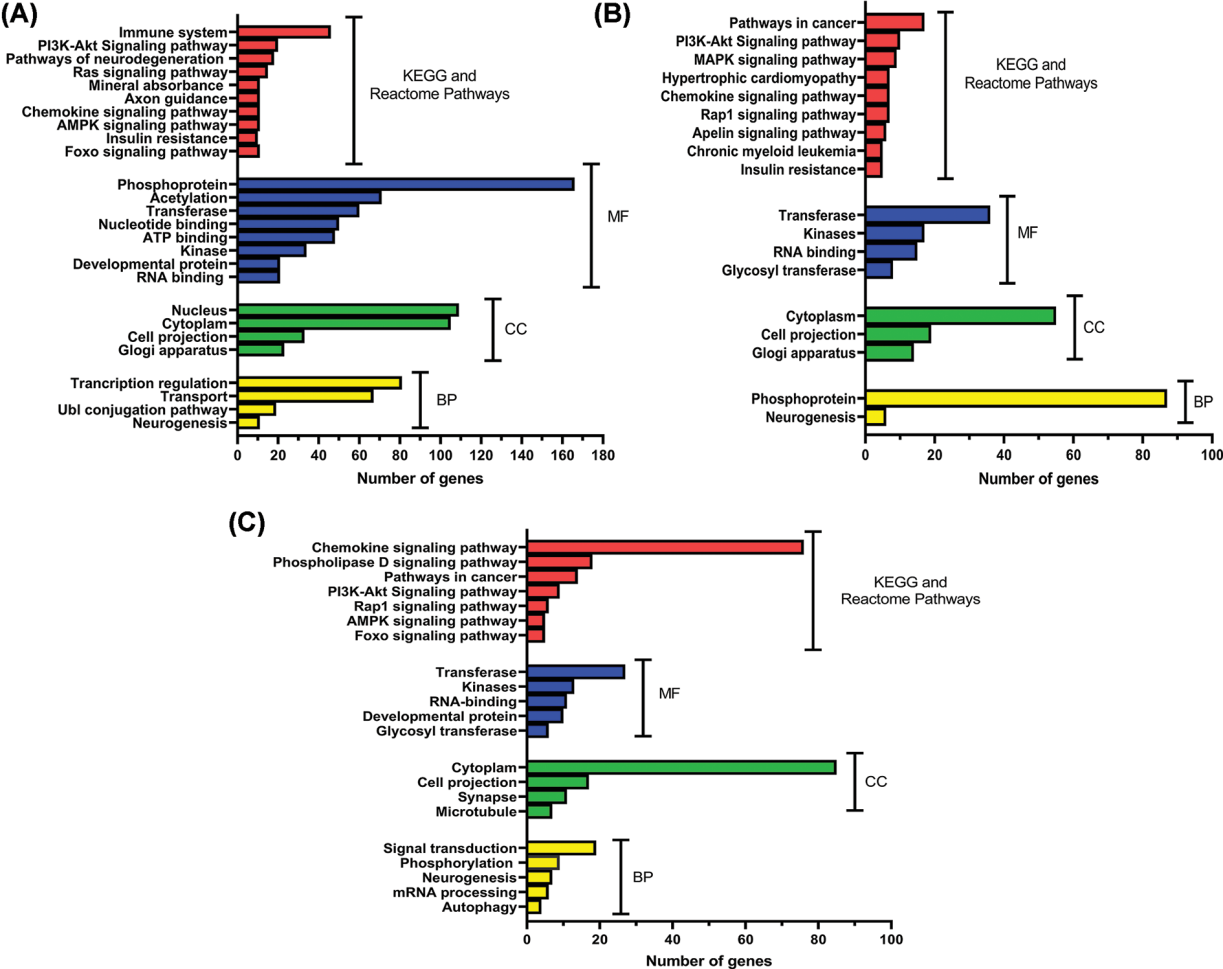
Gene ontology analysis for circ-CDC27 (A), circ-Med14 (B), and circ-TubD1(C) Bar graphs show the enriched GO terms of molecular function (MF), cellular components (CC), and biological process (BP) arranged according to the number of genes (*n*) involved. Results are shown only for *P* value match of < 0.001 and *n*≥5. CDC27, cell division cycle 27; KEGG, Kyoto Encyclopedia of Genes and Genomes; Med14, mediator complex subunit 14; TubD1, tubulin delta 1.

To get more insight into the cellular roles and patterns of regulation of the three circRNAs, the 515, 267, 210 overlapping predicted target genes of Circ-CDC-27, Circ-Med14, and Circ-TubD1, respectively, were assessed by using the functional enrichment tool David and ShinyGO [23,24] as shown in Figure 8. Circ-CDC27, Circ-Med14, and Circ-TubD1 target genes were involved in various molecular functions (MFs) activities such as acetylation, transferase, kinase activities, in addition to nucleotide, RNA, and ATP binding. In GO biological processes (BPs) enrichment, the 515 target genes of Circ-CDC27 were mainly enriched by the transcription regulation, ubiquitin-like proteins (Ubl) conjugation and neurogenesis pathways, while, Circ-Med14 target genes were mainly involved in neurogenesis. On the other hand, Circ-TubD1 target genes were enriched in neurogenesis in addition to signal transduction, phosphorylation, and autophagy. For cellular components (CCs), target genes of the circRNAs were commonly enriched by the cytoplasm, nucleus, and golgi apparatus.

To further explore the correlation between the circRNAs, diseases, and pathways, Wikipathways, Reactome, BioCarta, and Canonical pathways enrichment analysis were also performed by David and ShinyGO using each circRNAs’ target genes. The results showed that in the KEGG functional sets, PI3K-Akt, Rap1, pathways in cancer, AMPK, chemokine signaling pathways were the common pathways enriched. Moreover, circ-CDC27 target genes were mainly involved in immune system and neurodegeneration pathways.

## Discussion

SLE is an autoimmune disease that affects various systems in the body and exhibits diverse clinical manifestations. Increasing evidence emphasizes the significant role of cytokines in the development of SLE and their potential as biomarkers and targets for emerging therapies [1]. The most specific laboratory indicators for SLE remain autoantibodies, such as ANA and anti-dsDNA antibodies. Autoantibodies play a critical role in improving the diagnosis and prognosis of the disease. However, their sensitivity can vary based on the specific organ being targeted and the disease activity during the time of assessment [27]. In addition, the complexity of SLE pathogenesis and its varied symptoms often lead to misdiagnosis [1]. Therefore, it is crucial to identify new and distinctive biomarkers that can predict the early stages of SLE. This would enable accurate diagnosis, improved symptom management, and enhanced evaluation of medication effectiveness in clinical trials. To gain fresh insights into the mechanisms underlying SLE, we investigated the expression levels of novel biomarkers in SLE, aiming to unravel the mysteries surrounding SLE pathogenesis and contribute to early diagnosis and treatment approaches. In consequence, the differential expression levels of selected RNAs: circ-CDC27 (circ-0044235), circ-Med14 (circ-0140271), circ-TubD1 (circ-0044897), rno-miR-146a-5p, and TRAF6 were estimated in serum obtained from SLE-induced rats.

Previous studies revealed the biological and clinical relevance of epigenetic changes related to the development of SLE including alterations in DNA methylation, histone modification and non-coding RNAs expression [28–30]. In the current study, SLE rats demonstrated significantly higher expression levels of circ-CDC27 (circ-0044235), circ-Med14 (circ-0140271), rno-miR-146a-5p, and TRAF6, with significantly lower expression levels of circ-TubD1 (circ-0044897). CircRNAs act as ceRNAs by sponging miRNAs as previously demonstrated by Niu et al. [7] who reported that circ-TubD1 acted as miR-146a-5p sponge affecting the production of pro-inflammatory cytokines. Moreover, silencing circ-TubD1 has been reported to significantly down-regulate the expression of TRAF6 compared to the corresponding negative control in hepatic cell line. Lately, validation assays identified the decrease in the level of circ-0044235 in peripheral blood leukocytes of SLE patients; however, a significant increase in its level was detected in new-onset SLE which agrees with the results of our present study [12]. On the other side, the expression of circ-0140271 has been reported to be significantly higher in patients with RA than controls, providing evidence that it was likely to be used as a promising diagnostic biomarker for RA [15]. However, no study has previously demonstrated the expression of circ-0140271 in SLE.

MiRNAs have emerged as crucial players in SLE progression. Abnormalities in levels of miR-146a-5p in SLE patients have been previously published but with inconsistent results. Zhu et al. [10] and Zheng et al. [31] reported that miR-146a-5p expression level was decreased in SLE patients, which was contradictory to our results. They also proved that miR-146a-5p was identified as an epigenetic regulator of TRAF6 expression in SLE patients suffering from lupus nephritis which led to an increase in the expression level of TRAF6 supporting our findings. On the other hand, other studies by Shumnalieva et al. [32], Tawfik et al. [33], Labib et al. [34] and El-Akhras et al. [35] reported increased level of miR-146a-5p in SLE patients, which agrees with our results [11,36].

In the current study, data of ROC curve analysis of the circRNAs: circ-CDC27 (circ-0044235), circ-Med14 (circ-0140271), circ-TubD1 (circ-0044897), and rno-miR-146a-5p suggests that they could be useful as SLE diagnostic biomarkers. Furthermore, our correlation study revealed a negative association between circ-TubD1 expression level and rno-miR-146a-5p, while rno-miR-146a-5p was positively correlated with TRAF6. These associations suggest that the elevated expression of TRAF6 may be attributed to the action of ceRNAs, a novel mechanism of gene regulation that facilitates the dysregulated expression of both miRNAs and mRNAs. The decrease in circ-TubD1 expression in SLE-induced rats indirectly enhances the expression of TRAF6 by facilitating the release of rno-miR-146a-5p. This is supported by the identification of TRAF6 as a target for miR-146a-5p in lupus nephritis by Zhu et al. [10] and Zheng et al. [31]. These results provide some insights into the potential involvement of circ-TubD1 in the pathogenesis of SLE. However, additional investigations employing high-throughput gene-silencing techniques are necessary to assess the functional aspects and establish the interconnected relationships among the three RNA types within the proposed ceRNA network.

To further predict the main pathways in which circ-CDC27, circ-Med14, and circ-TubD1 are involved, a series of bioinformatics analysis was done using several databases. We created a process that combines functional analysis with in-silico identification. First, the target miRNAs were obtained from Targetscan [20], miRanda V3.3a, and CircAtlas [19], then the common miRNAs were chosen for further analysis. The target genes of the chosen miRNAs were then predicted using Targetscan [20], miRDB [21], and pictar [22]. The interaction network was visualized using Cytoscape V3.10. The 515, 267, 210 overlapping genes of circ-CDC27, circ-Med14, and circ-TubD1 were then submitted to David [23] and ShinyGO [24] for GO annotation and functional enrichment analyses.

Circ-CDC27, circ-Med14, and circ-TubD1 target genes were found to be involved in various MFs’ activities such as acetylation, transferase, kinase activities. Previously, acetylation was one of the epigenetic factors contributing to SLE [37,38]. Moreover, previous studies reported that altered transferases activity as glutathione S, gamma glutamyl, and nicotinamide phosphoribosyl transferases were associated with SLE [39–41]. In BPs enrichment, the target genes of circ-CDC27 were mainly enriched by the transcription regulation, Ubl conjugation, and neurogenesis pathways, while, circ-Med14 target genes were mainly involved in neurogenesis. On the other hand, circ-TubD1 target genes were enriched in neurogenesis in addition to signal transduction, phosphorylation, and autophagy. For CCs, target genes of the circRNAs were commonly enriched by the cytoplasm, nucleus, and golgi apparatus.

To further explore the correlation between the circRNAs, diseases, and pathways, Wikipathways, Reactome, BioCarta, and Canonical pathways enrichment analysis were also performed by David and ShinyGO using each circRNAs’ target genes. The results showed that in the KEGG functional sets, PI3K-Akt, Rap1, pathways in cancer, AMPK, and chemokine signaling pathways were the common pathways enriched. Studies have shown that activation of the PI3K/Akt/mTOR and rap 1 pathways in lymphocytes alters the development of systemic autoimmunity, establishing a connection between this pathway and autoimmune diseases [42–44]. Besides, Lai et al. [45] found that activating mTOR triggered IL-4 formation and necrotic death of T cells in patients with SLE. The immunopathogenesis of SLE is an intricate process that relies on the interplay and synergistic impact of multiple cytokines, chemokines, and signaling molecules [46]. Moreover, miR-125a was reported to elevate the level of the inflammatory chemokine by targeting KLF13 in SLE [47].

Circ-CDC27 target genes were found to be mainly involved in the immune system and neurodegeneration pathways. The development of SLE involves a range of immunological abnormalities, including dysregulation of both innate and adaptive immune responses [48,49]. Our study showed that the target genes of circ-Med14 were part of the MAPK and apelin signaling pathways, in addition to the progression of insulin resistance, hypertrophic cardiomyopathy, and chronic myeloid leukemia (CML). Previous research has suggested that the aberrant activation of p38 MAPK is implicated in the inflammation observed in SLE, which can result in the progressive development of lupus nephritis and autoimmune hepatitis, causing damage to tissues and organs [50]. Hsa-circ-0123190 level decreased in lupus nephritis and acts by sponging hsa-miR-483-3p, which was validated to interact with the apelin receptor (APLNR) [51]. SLE was correlated with increased insulin resistance, cardiac disorders, and CML [52–54]. On the other hand, circ-TubD1 was predicted to be involved in phospholipase D and foxo signaling pathways. Phospholipase D2 gene mutation was associated with familial SLE [55]. Numerous more findings demonstrated the importance of FOXO3a in the etiology of autoimmune disorders, such as SLE [56,57].

In conclusion, the significant contribution of our study lies in the introduction of novel biomarkers; circRNAs; circ-TubD1 (circ-0044897), circ-CDC27 (circ-0044235), and circ-Med14 (circ-0140271), miRNA (rno-miR-146a-5p), and mRNA (TRAF6) that may play a potential role in the development of SLE. Besides, the present study provides bioinformatic analysis of the circRNAs providing a multi-level gene regulatory network that involves interactions among circRNA, miRNA, and mRNA associated with SLE pathogenesis and indicating their involvement in various processes and pathways related to SLE pathogenesis and their potential as a diagnostic and therapeutic target in the future. Further studies are needed in different animal models to strengthen the validity and translational relevance of our findings, advancing our understanding of circ-TubD1, circ-CDC27, circ-Med14, rno-miR-146a-5p, and TRAF6 as potential diagnostic markers in SLE. Moreover, their prognostic value in human subjects, treatment response prediction, and potential as therapeutic targets for SLE should be investigated in the future.

## Supplementary Material

Supplementary Figure S1 and Tables S1-S2

## Data Availability

The datasets generated during and/or analyzed during the current study are available from the corresponding author.

## Abbreviations

ANA: antinuclear antibodies
CML: chronic myeloid leukemia
IFA: incomplete Freund’s adjuvant
SLE: systemic lupus erythematosus
TNFR: tumor necrosis factor receptor
TRAF6: TNFR-associated factor 6
TubD1: tubulin delta 1
