# Supplementary material for: Functional analysis of a panel of molecular markers for diagnosis of systemic lupus erythematosus in rats
Source: Biosci Rep. 2024 Jul 19;44(7):BSR20240318. doi: 10.1042/BSR20240318 (PMC11263041; doi:10.1042/BSR20240318)
Supplement: Supplementary Figure S1 and Tables S1-S2 [file BSR-2024-0318_supp.pdf]

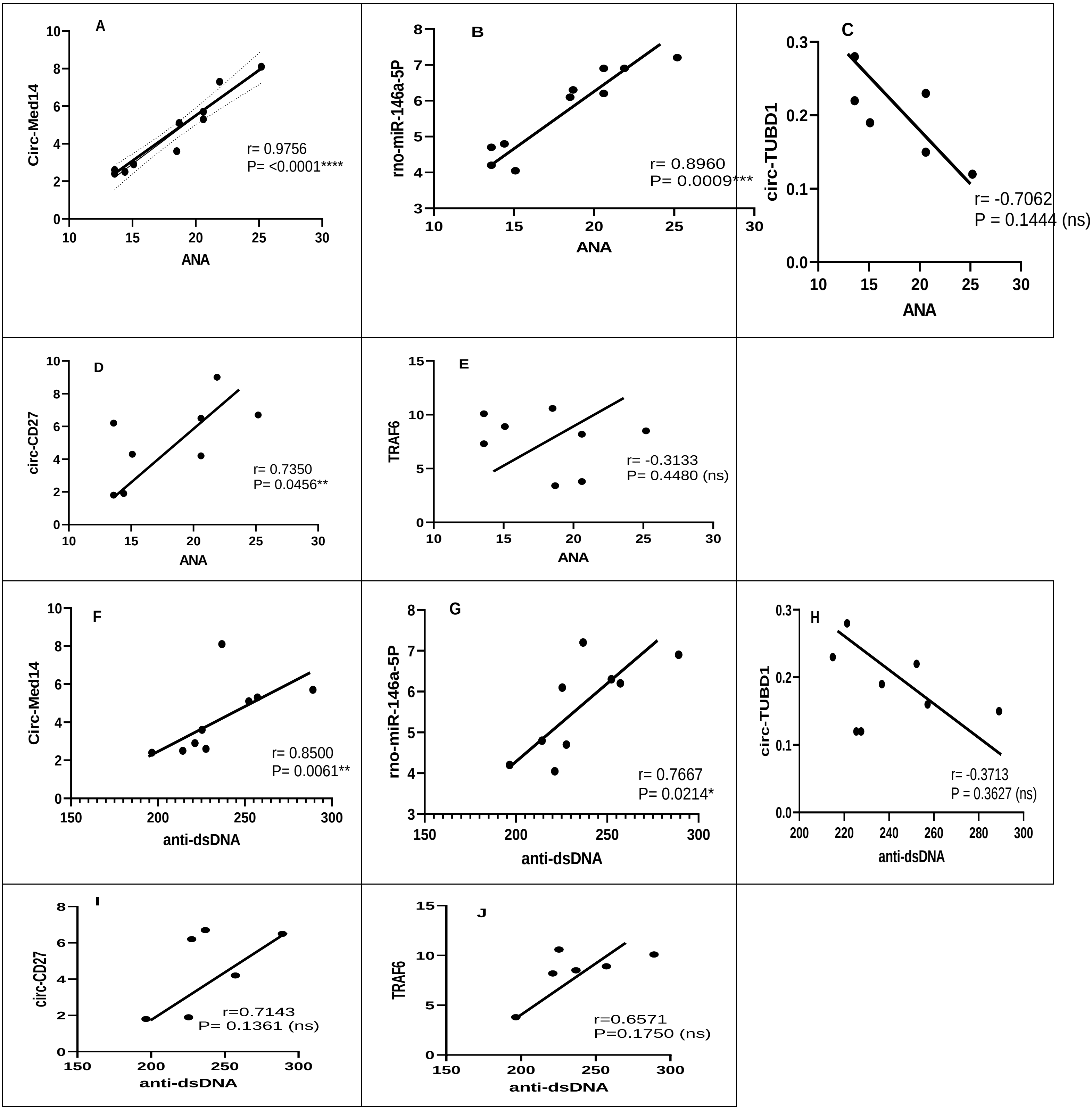


**Supplementary Figure 1: Pearson correlation between ANA levels with (A): circ-Med14, (B): rno-miR-146a-5p, (C): circ-TUBD1, (D): circ-CD27, (E): TRAF6 and between anti-dsDNA with (F): circ-Med14, (G): rno-miR-146a-5p, (H): circ-TUBD1, (I): circ-CD27, (J): TRAF6 levels.**

**Table S1: Concentration levels of ANA and anti-dsDNA antibody measured in sera of SLE-induced rats**

| Parameter | Control group | SLE-induced group |
|---|---|---|
| ANA (U/l) | 6.61 ± 2.504 | 18.81 ± 3.701*** |
| Anti-dsDNA antibody (U/l) | 104 ± 13.5 | 234 ± 26.3*** |

Data are represented as mean ± S.D. ANA: anti-nuclear antibody, anti-dsDNA: anti-double stranded DNA. Significantly different at ***P < 0.001.

**Table S2: Expression levels of the studied RNAs measured in sera of SLE-induced rats**

| Parameter | Control group | SLE-induced group |
|---|---|---|
| **Circ-CDC27 (Circ-0044235)** | **1.013 ± 0.01** | **5.08 ± 2.5*** ** |
| **Circ-Med14 (Circ-0140271)** | **1.018 ± 0.016** | **4.42 ± 2.16*** ** |
| **Circ-TubD1** | **1.036 ± 0.02** | **0.35 ± 0.17*** ** |
| **Rno-miR-146a-5p** | **1.024 ± 0.018** | **4.58 ± 1.21*** ** |
| **TRAF6** | **1.016 ± 0.014** | **7.6 ± 2.68*** ** |

Data are represented as mean ± S.D. CDC27: cell division cycle 27, Med14: mediator complex subunit 14, Traf6: tumor necrosis factor receptor-associated factor 6, TubD1: tubulin delta 1. Significantly different at ***P < 0.001.
